# Differences in contraceptive prices between and within family planning outlets in urban and semiurban Nigeria: a prospective cohort study

**DOI:** 10.1016/j.xagr.2022.100131

**Published:** 2022-11-05

**Authors:** Claire W. Rothschild, Bo Hu, Justin Archer, Ekerette Emmanuel Udoh, Chinedu Onyezobi, Anthony Nwala, CM4FP Group.

**Affiliations:** a Population Services International, Washington, DC (Dr Rothschild); b Care Policy and Evaluation Centre, London School of Economics and Political Science, London, United Kingdom (Dr. Hu); c Independent Consultant, Washington, DC (Mr. Archer); d and Society for Family Health Nigeria, Abuja, Nigeria (Mr. Udoh, Mr. Onyezobi, and Dr. Nwala)

**Keywords:** contraception, family planning, market, pricing, supply-side

## Abstract

**BACKGROUND:**

There is a lack of comprehensive evidence assessing variability and volatility in contraceptive prices. Improved understanding of contraceptive pricing, both between and within public and private service delivery points situated within complex, mixed health systems, may improve understanding of contraceptive access from the perspective of the consumer.

**OBJECTIVE:**

To describe variability and volatility in contraceptive method prices within localized urban and semiurban markets in Nigeria.

**STUDY DESIGN:**

We used product audit data from a complete census and longitudinal cohort of family planning vendors within 4 urban and semiurban study sites in Nigeria. Differences in outlet-level minimum prices by outlet type were assessed using generalized estimating equations. We presented descriptive summaries of within-outlet changes in minimum price over time.

**RESULTS:**

Among 672 family planning vendors, outlet-level minimum prices were significantly higher in private facilities/outlets than in public facilities. The outlet-level minimum price was $9.4 (95% confidence interval, $5.7–$13.2) higher for implants in private vs public facilities. We observed high availability of free contraceptive products in the public sector (79%–100%), moderate levels for specific contraceptive product types among community health workers and private facilities (28%–62% for male condoms), and low prevalence among private nonfacility outlets (0%–3%). Variability in contraceptive prices was high within private facilities and nonfacility outlets: standard deviations in the distribution of long-acting reversible contraceptive products ranged from $9.7 for implants to $13.1 for intrauterine devices in the private sector. Changes in minimum prices by contraceptive method type were common within the same outlets over time in the private sector.

**CONCLUSION:**

We observed high variability between and within contraceptive vendors in selected Nigerian family planning markets. Further research assessing the impact of price variability is critical for understanding contraceptive access and decision-making from the consumer's perspective.

**AJOG Global Reports at a Glance**

**Why was this study conducted?**

Using data from the Consumer's Market for Family Planning study, a multicountry study that assessed contraceptive supply and demand, we aimed to understand the extent of pricing variability and volatility in contraceptive commodities in several urban and semiurban Nigerian settings.

**Key findings**

Availability of free contraceptive products was high in public and moderate (for specific contraceptive methods) in private facilities, but low in private nonfacility outlets. Within-facility changes in minimum prices were common over 3 quarterly waves of data collection.

**What does this add to what is known?**

In addition to the well-documented large public–private differences in pricing of contraceptive commodities, this study found that family planning consumers in urban and semiurban markets in Nigeria face complex markets characterized by variable pricing within the private sector and volatility in minimum prices over short time periods.

## Introduction

There is a lack of comprehensive evidence assessing variability and volatility in contraceptive prices from the perspective of the healthcare consumer. Evidence on the impact of costs on contraceptive behaviors is mixed,^1^ with numerous methodological challenges, including the relative lack of experimental evidence and the complexity of non-cost–related factors that influence contraceptive demand. Although several previous studies suggest that demand for contraception is relatively cost-insensitive,^2^, ^3^, ^4^ robust evidence is limited. Socioeconomic gradients in the likelihood of long-acting reversible contraceptive (LARC) use in sub-Saharan Africa indicate that cost barriers may be prohibitive for the poorest women, particularly for LARC methods that may include fees for provider insertion and removal in addition to the commodity itself.^5^^,^^6^ There is substantial regional variability in the proportion of women with unmet need for contraception who cite lack of access or high cost as a reason for nonuse: an analysis of the Demographic and Health Surveys (DHS) Program found that only 4% to 8% of women cited lack of access as a reason for nonuse overall, although this proportion was as high as 15% in Middle Africa and 10% in West Africa.^7^

Although contraceptive services are often subsidized or free in the public sector in low- and middle-income countries (LMIC), the private sector, and in particular pharmacies and drug shops, play an increasingly important role in family planning (FP) service delivery. In urban areas of Nigeria and Kenya, for example, most women using oral contraceptive pills (OCPs), emergency contraceptive pills (ECPs), and condoms reported obtaining the method from a drug shop or pharmacy.^8^

Financial barriers may be particularly salient for young and unmarried women, many of whom have strong preferences for accessing contraception through private outlets. A study of women in urban Nigeria and Kenya found that young Kenyan women (<25 years old) and unmarried women in both countries were significantly more likely to report obtaining contraception from a pharmacy or drug shop than from a facility.^8^ Pharmacies and drug shops are often preferred because of privacy concerns, convenience, fast service, and perceived youth-friendliness.^9^, ^10^, ^11^ In a mixed-methods study in Kenya, young people identified pharmacies as a preferred option that afforded privacy without the prohibitive costs of private facilities; many young people were willing to pay more for private pharmacy services to access high-quality, private care, citing pharmacies’ consistent pricing as an added benefit.^10^

Despite the key role of the private sector in contraceptive provision in urban LMIC settings, there is relatively little comprehensive evidence assessing variability in the cost of contraceptive commodities between different outlets and within the same outlets over time. Such evidence is important for understanding costs of contraceptive services from the perspective of a consumer navigating a complex total FP market. We used data from the Consumer's Market for Family Planning (CM4FP) study, which conducted a complete census of all FP outlets in selected study sites and longitudinal follow-up of identified outlets in Kenya, Nigeria, and Uganda. We used data from 4 urban and semiurban sites in Nigeria to assess variability in contraceptive commodity pricing within these markets. We described differences in outlet-level prices of each contraceptive method type between outlets and changes within outlets over time, with a focus on outlet-level minimum prices to explore implications for contraceptive access.

## Materials and Methods

### Study population

The CM4FP study was conducted in purposively sampled urban and semiurban (and 1 rural) settings in Kenya, Nigeria, and Uganda from 2019 to 2020. The study methodology has been reported previously.^12^ Briefly, the study used a ring-fence census approach, which comprised a complete census and product audit of all outlets and community health workers (CHWs) offering FP methods or services (beyond just providing male condoms) within a specific geographic area (the outer ring). Outlet data were captured longitudinally for 3 quarters in Kenya and Nigeria and 2 quarters in Uganda (because of COVID-19–related suspensions to fieldwork).

In this analysis, we used longitudinal data captured over 3 quarterly survey rounds from 4 study sites in Nigeria: a 7.3-km^2^ large urban site in Lagos State, a 22.3-km^2^ medium urban site in Kaduna State, a 5-km^2^ small urban site in Abia State, and a 29.4-km^2^ semiurban site in Niger State. Specific details of the study site locations are withheld to ensure participant privacy, per study protocol. At the state level, the study sites are socioeconomically and culturally diverse: the percentage of the population in the lowest national wealth quintile varies from 0% in Lagos and Abia states and 6% in Kaduna State, to 17.4% in Niger State.^13^ Similarly, median years of education among women of reproductive age (15–49 years) ranges from >11 years in Lagos and Abia to 3 and 0 years in Kaduna and Niger states, respectively.^13^ Modern contraceptive use among women of reproductive age is most prevalent in Lagos State (29%), dropping to 14% in Kaduna, 11% in Abia, and 6% in Niger State.^13^

### Data sources

Each outlet survey included a product audit, in which price and other information (including wholesale/supplier cost, stockout history, and sales volumes) was collected for each unique contraceptive product (identified by brand and product type) in stock on the date of the audit. Unique products were audited once per outlet per round, with data on each available product recollected in each round of data collection. Therefore, each observation in the product audit data represents a unique contraceptive product offered within the specific outlet for a given round of data collection.

### Statistical analysis

To assess price variability, we combined all 3 survey rounds and retained data captured at the earliest (chronological) audit of each outlet. We retained outlet audit information for the first observation of the outlet only to avoid double-counting of products within outlets over time and to provide a cross-sectional snapshot of all outlets interviewed at their first survey round. For this analysis, we retained multiple products (ie, brands and specific product names within brands) if >1 product per contraceptive method type was stocked at the outlet. We excluded observations for 2 audited ECP products with high outlying recorded prices of 15,000 Nigerian naira (NGN) (∼$42 US dollars [USD]) given that the next highest ECP price was 3000 NGN (∼$8.4 USD). Prices in the main text are expressed in USD per unit on the basis of a contemporaneous midpoint conversion rate of 0.0028 NGN to 1 USD. All tables and figures are available in NGN in the Online Supplement. We presented descriptive summaries of the number of unique products, distribution of posted prices, and the proportion of audited products posted as free for each method type, by facility type (facility or nonfacility outlet, including proprietary patent medicine vendors [PPMVs] and pharmacies) and governing authority (public or private).

To assess differences in pricing between outlets, we used data from all survey rounds, with repeated observations of outlets over 3 quarters at most. We fit linear generalized estimating equation (GEE) models of outlet-level median prices for each contraceptive method type to assess the presence of statistically significant differences in average outlet-level minimum prices by outlet level and governing authority. We chose to focus on minimum prices at the outlet level because this best represents the minimum financial barriers experienced by would-be consumers. Multivariable linear GEE models were specified with an independent working correlation structure accounting for repeated observations at the outlet level, robust standard errors, and included outlet type and governing authority (public facility, private facility, private nonfacility outlet, or CHW); audit round (first–third); and an interaction term between outlet type and survey round as independent categorical variables.

To describe price differences within outlets over time, we presented descriptive summaries of changes in minimum price over time and estimated the proportion of outlets with increases in median and minimum prices over time, by method type.

### Ethics statement

The study protocol was reviewed and approved by the Population Services International Research Ethics Board (01.2019 and 04.2019) and the National Health Research Ethics Committee of Nigeria (NHREC/01/01/2007-27/05/2019). Informed consent was obtained before all study procedures. In Nigeria, verbal informed consent was obtained from all FP outlets and CHWs to protect participants and minimize risks to confidentiality.

## Results

The CM4FP study data from sites in Nigeria comprise audit information on a total of 672 unique FP outlets captured over 3 rounds of audits. For analyses of price variability and availability of free contraceptive commodities, we used an analysis sample comprising audit data for 2437 products captured from the first audit conducted within each outlet. Sample sizes of audited products ranged from 1307 male condoms to 51 intrauterine devices (IUDs) (Table 1).

**Table 1:** Count of audited products by method type and outlet characteristics

Service delivery point type	Male condom (n)	OCP (n)	ECP (n)	Injectable (n)	Implant (n)	IUD (n)	Total (n)
Public facility (n=34)	24	34	0	69	32	15	174
Private facility (n=78)	31	23	4	41	30	26	155
Private outlet (n=425)	1229	303	379	67	4	0	1982
Public CHW (n=42)	4	5	1	17	3	1	29
Private CHW (n=89)	20	20	6	28	14	4	92
Hybrid CHWa (n=4)	1	0	0	2	2	0	5
Total (n=672)	1309	385	392	222	85	46	2437

*CHW*, community health worker; *ECP*, emergency contraceptive pill; *IUD*, intrauterine device; *OCP*, oral contraceptive pill.

^a^Hybrid CHWs reported being affiliated with both public and private facilities. *Notes:* Counts represent the sum of all audited products, per contraceptive method type, collected at each unique service delivery point (with data for only 1 audit per service delivery point, even if multiple audits were conducted).

We observed differences in availability of free products and in posted prices among nonfree products by outlet type and governing authority (Table 2). Most contraceptive commodities audited in public facilities were posted as free. In round 1, for example, the proportion of free products in public facilities ranged from 100% for audited OCPs and 96% for male condoms to 83% of IUD products (Table 1). We found similar results in survey rounds 2 and 3, with the lowest proportion of free products by method observed for implants in round 3 (at 79%).

**Table 2:** Median prices by method type and outlet type, survey rounds 1 to 3 (US dollar per unit)

Contraceptive method	Round 1				Round 2				Round 3			
	Public facility	Private facility	Outlet	CHW	Public facility	Private facility	Outlet	CHW	Public facility	Private facility	Outlet	CHW
Male condom												
Number of product	23	37	1163	25	21	33	1379	43	28	39	1435	—
Median prices (excluding free products)	0.09	0.09	0.14	0.09	—	0.09	0.14	0.09	—	0.09	0.14	—
% of free product	96%	54%	0.1%	28%	100%	48%	0.07%	58%	100%	62%	0.07%	—
OCP												
Number of product	34	25	293	25	49	43	257	51	59	43	169	—
Median prices (excluding free products)	—	1.17	0.28	0.31	0.28	0.56	0.28	0.42	0.25	0.84	0.28	—
% of free product	100%	28%	0.3%	20%	96%	23%	0.39%	62%	93%	21%	0.59%	—
ECP												
Number of product	—	6	360	7	—	6	403	4	—	8	431	—
Median prices (excluding free products)	—	0.84	1.12	0.7	—	1.33	0.98	0.49	—	0.7	0.98	—
% of free product	—	17%	0%	0%	—	0%	0%	0%	—	0%	0%	—
Injectable												
Number of product	70	46	65	45	83	47	67	81	85	49	76	—
Median prices (excluding free products)	0.56	1.68	1.4	0.84	0.84	4.2	1.1	0.84	0.56	2.0	1.4	—
% of free product	89%	17%	3.10%	36%	89%	11%	1.50%	53%	84%	10%	1.32%	—
Implant												
Number of product	34	40	4	19	44	40	7	18	43	31	8	—
Median prices (excluding free products)	1.4	5.6	1.96	2.8	2.8	7	2.2	2.8	2.8	7	2.2	—
% of free product	88%	7.50%	0%	21%	84%	7.5%	0%	39%	79%	13%	13%	—
IUD												
Number of product	18	33	—	5	20	32	3	6	14	29	3	—
Median prices (excluding free products)	1.4	7	—	1.96	2.8	7	3.4	2.8	2.8	7.7	4.2	—
% of free product	83%	3%	—	20%	80%	6.3%	0%	50%	82%	3.5%	0%	—

*CHW*, community health worker; *ECP*, emergency contraceptive pill; *IUD*, intrauterine device; *OCP*, oral contraceptive pill.

Free products were less common in the private sector, particularly for LARC methods. In round 1, only 7.5% and 3% of implants and IUDs offered in private facilities were free, respectively, as opposed to 88% of implants and 88% of IUDs in public facilities. Of a total of 4 observations of audit data captured on implants in round 1 in private outlets, none were free. Free products were more prevalent for short-acting reversible method products offered in private facilities (in round 1: 54% for male condoms, 28% for OCPs, and 17% for injectables). Free products were least common in private outlets (such as pharmacies and drug shops), with no instances of free ECPs and <1% of both male condoms and OCPs across all survey rounds.

**Figure 1:**
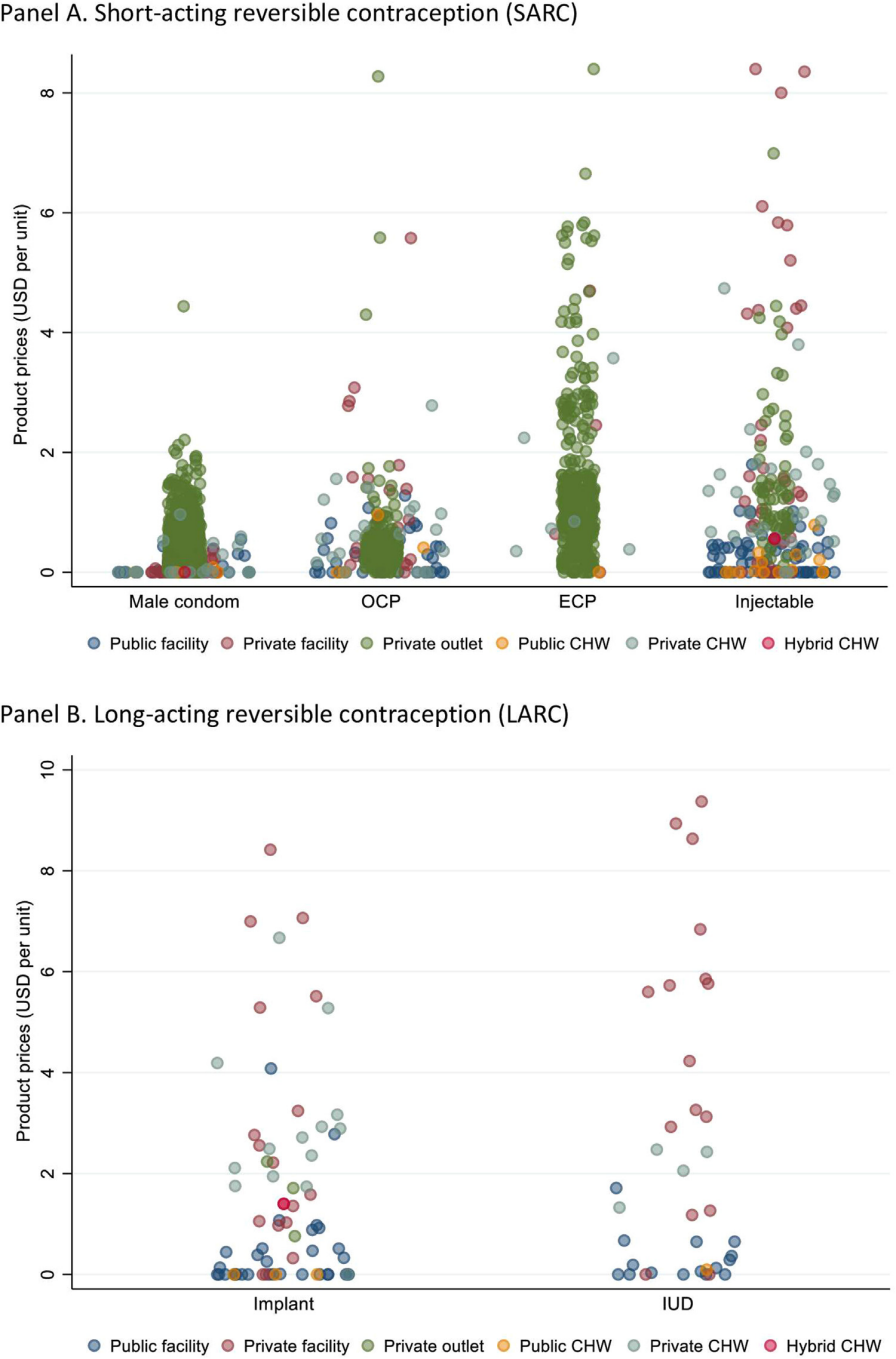
Price of audited contraceptive methods, by method and outlet type. A, short-acting reversible contraception; B, long-acting reversible contraception.

Within private facilities and nonfacility outlets, we observed high price variability within products of the same contraceptive method type. For example, we estimated standard deviations of $13 USD and $9.7 USD in the distribution of IUD and implant prices, respectively, in the private sector (Figure 1). We observed little variability in the public sector, given the high availability of free products: we estimated standard deviations of $0.51 USD and $0.69 USD in the distributions of IUD and implant prices among public sector facilities.

Rothschild. Differences in contraceptive prices between and within family planning outlets in urban and semiurban Nigeria. Am J Obstet Gynecol Glob Rep 2022.*CHW*, community health workers; *ECP*, emergency contraceptive pills; *IUD*, intrauterine device; *OCP*, oral contraceptive pill.

We observed statistically significant differences in outlet-level minimum prices of contraceptive commodities between outlets of different types, with the largest differences observed for injectable and LARC methods offered in private vs public facilities (Table 3). Relative to public facilities, average outlet-level minimum price was $3.5 (95% confidence interval [CI], 1.8–5.2) higher for injectables and $9.4 (95% CI, 5.7–13.2) higher for implants in private facilities, and $1.5 (95% CI, 1.2–1.9) and $3.9 (95% CI, −0.3 to 8.0) higher in private outlets, respectively. We observed similar patterns for short-acting methods, but with differences in price of smaller absolute magnitude: for example, mean minimum price of male condoms was $0.09 (95% CI, 0.07–0.10) higher in private outlets, and $0.05 (0.02–0.08) higher when provided by CHWs, after adjustment for timing of survey round.

**Table 3:** Generalized estimating equation model of outlet-level minimum prices for contraceptive methods, by method type

Independent variable	Condomβ (SE)	ECPβ (SE)	Injectableβ (SE)	Implantβ (SE)
Public facility	Ref.	—	Ref.	Ref.
				
Private facility	0.026	Ref.	3.491^a^	9.430^a^
	−0.01		−0.87	−1.92
Private outlet	0.087^a^	−0.596	1.515^a^	3.85
	−0.01	−0.74	−0.18	−2.11
CHW	0.054^a^	−0.568	0.664^a^	1.980^a^
	−0.02	−0.81	−0.15	−0.46
Round 1	Ref.	Ref.	Ref.	Ref.
				
Round 2	−0.004	0.562	0.071	0.268
	0	−0.77	−0.09	−0.18
Round 3	−0.004	−0.574	−0.013	0.205
	0	−0.66	−0.04	−0.2
Public facility # Round 1	Ref.	—	Ref.	Ref.
				
Public facility # Round 2	Ref.	—	Ref.	Ref.
				
Public facility # Round 3	Ref.	—	Ref.	Ref.
				
Private facility # Round 1	Ref.	Ref.	Ref.	Ref.
				
Private facility # Round 2	−0.005	Ref.	−0.593	−2.256
	−0.02		−0.76	−2.06
Private facility # Round 3	−0.001	Ref.	−0.725	−3.246
	−0.02		−0.86	−1.95
Private outlet # Round 1	Ref.	Ref.	Ref.	Ref.
				
Private outlet # Round 2	−0.007	−0.758	−0.568^b^	2.625
	−0.01	−0.78	−0.21	−2.48
Private outlet # Round 3	0.01	0.387	0.056	−1.185
	−0.01	−0.67	−0.14	−1.96
CHW # Round 1	Ref.	Ref.	Ref.	Ref.
				
CHW # Round 2	−0.028	−1.202	−0.253	−1.116
	−0.02	−0.84	−0.19	−0.63
CHW # Round 3	—	—	—	—
				
Constant	0.004	1.568^c^	0.088^c^	0.14
	0	−0.74	−0.04	−0.09

Separate multivariable linear generalized estimating equation (GEE) models were fit to estimate outlet-level median price for each contraceptive method type (indicated by the column headers). GEE models were specified with an independent working correlation structure, robust standard errors, and clustering within outlet. “#” notation indicates interaction terms between outlet type and survey round. β coefficient was interpreted as the mean difference in price per contraceptive unit.

*β*, beta coefficient from the linear model; *CHW*, community health worker; *ECP*, emergency contraceptive pill; *Ref.*, referent category; *SE*, robust standard error.

a *P*<.001

b *P*<.01

c *P*<.001.

In aggregate, there was little change in mean outlet-level minimum prices across survey rounds after adjustment for outlet-level characteristics (type and governing authority): we estimated few statistically significant differences in minimum prices in rounds 2 and 3 relative to the first round of data collection. However, changes over time in outlet-level minimum and median prices were common in the private sector (Table 4). From the second to the third audit, for example, most private facilities posted increased minimum prices for OCPs (67%) and injectables (62%), whereas most private outlets reported increased minimum prices for male condoms (86%), OCPs (81%), ECPs (67%), and injectables (72%). Increases in minimum prices in the public sector were rare over the same time period, with no facilities with observed increases in minimum prices of condoms and ≤10% of facilities with observed increases in minimum prices for OCPs, injectables, and implants.

**Table 4:** Proportion of outlets exhibiting a change in median price (of any magnitude) since the most recent survey round

Panel A. Outlet audit, round 2					
	Male condom	OCP	ECP	Injectable	Implant
Among facilities stocking method:	n (%)	n (%)	n (%)	n (%)	n (%)
Public facility					
Increase in minimum price	0 (0)	0 (0)	—	2 (7)	2 (12)
Increase in median price	0 (0)	0 (0)	—	2 (7)	2 (12)
Private facility					
Increase in minimum price	2 (13)	1 (6)	3 (100)	8 (32)	6 (33)
Increase in median price	2 (13)	1 (6)	3 (100)	9 (36)	6 (33)
Private outlet					
Increase in minimum price	71 (25)	37 (22)	49 (28)	12 (43)	0 (0)
Increase in median price	95 (33)	40 (24)	65 (37)	11 (39)	0 (0)
CHW					
Increase in minimum price	0 (0)	5 (50)	0 (0)	5 (31)	1 (33)
Increase in median price	0 (0)	5 (50)	0 (0)	5 (31)	1 (33)
Panel B. Outlet audit, round 3					
Public facility					
Increase in minimum price	0 (0)	1 (4)	—	3 (10)	2 (10)
Increase in median price	0 (0)	1 (4)	—	3 (10)	2 (10)
Private facility					
Increase in minimum price	2 (20)	16 (67)	0 (0)	13 (62)	5 (33)
Increase in median price	2 (20)	16 (67)	1 (33)	13 (62)	4 (27)
Private outlet					
Increase in minimum price	287 (86)	88 (81)	132 (67)	23 (72)	1 (20)
Increase in median price	273 (82)	80 (73)	123 (62)	24 (75)	1 (20)

Change in each survey round estimated as difference in outlet-level minimum price between the current and most recent survey round at the outlet level; CHWs were not included in round 3 of data collection.

*CHW*, community health worker; *ECP*, emergency contraceptive pill; *OCP*, oral contraceptive pill.

**Figure 2:**
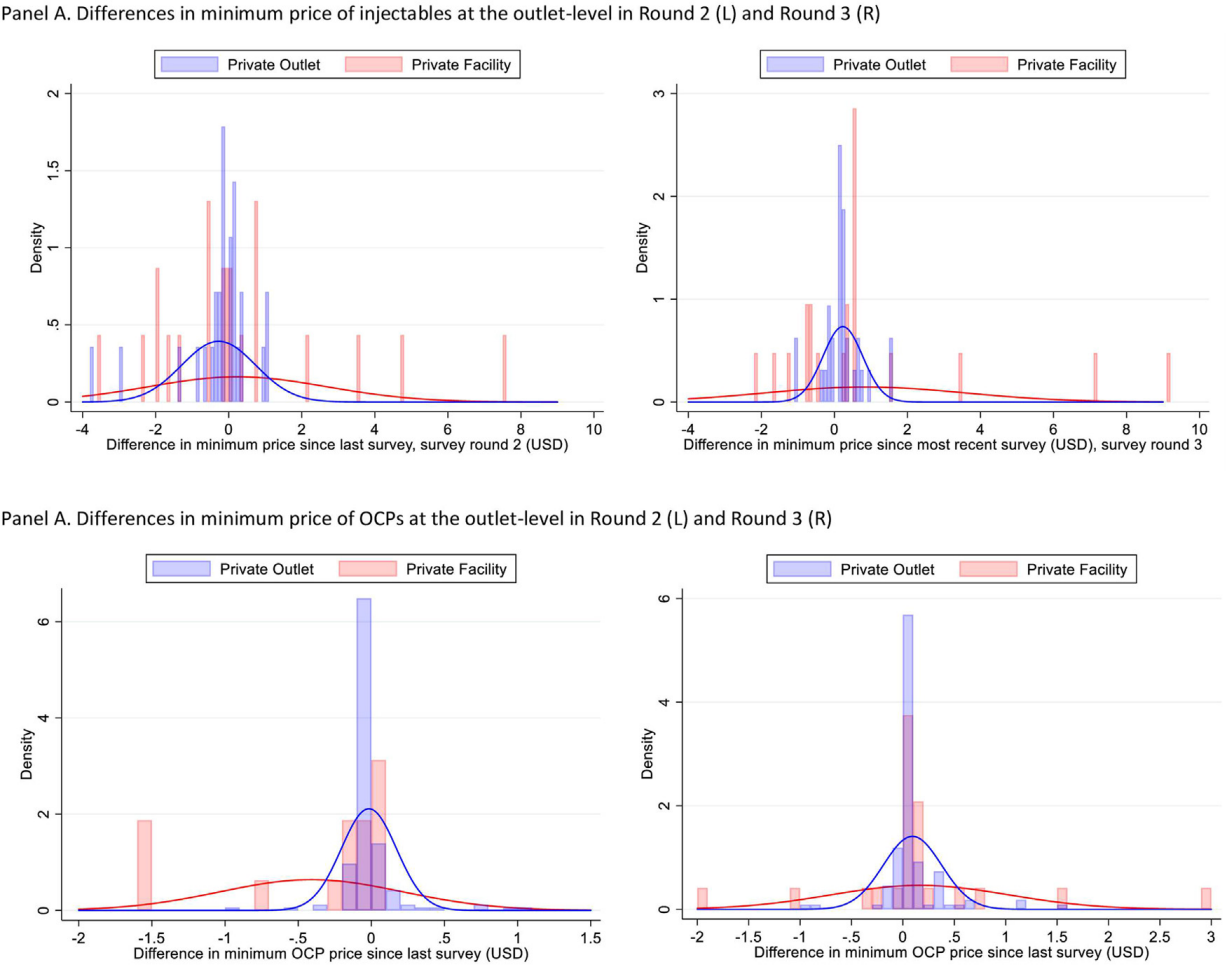
Distribution of changes in outlet-level minimum prices since prior survey round. **A,** Differences in minimum price of injectables at the outlet-level in Round 2 (L) and Round 3 (R). **B,** Differences in minimum price of OCPs at the outlet-level in Round 2 (L) and Round 3 (R).

Descriptive comparisons of the distribution of changes in minimum prices within outcomes over time suggested higher volatility in private facilities compared with private nonfacility outlets for injectables and OCPs (Figure 2). We observed similar descriptive patterns for implants and ECs (data not shown).

Rothschild. Differences in contraceptive prices between and within family planning outlets in urban and semiurban Nigeria. Am J Obstet Gynecol Glob Rep 2022.*OCP*, oral contraceptive pill.

## Discussion

There are relatively little detailed longitudinal data on pricing and availability of contraceptive products and services provided by the full range of providers—from large public hospitals to small private drug shops—that operate within complex healthcare markets in urban LMIC settings. Using data from CM4FP, we examined variability and differences in pricing of contraceptive products between and within FP outlets over time in 3 urban and 1 semiurban site in Nigeria. We observed substantial differences in price for specific contraceptive method types between outlets of different types, with higher prices and fewer free products consistently observed among private facilities, private outlets, and CHWs, relative to public facilities.

We also observed price differences in contraceptive methods of the same type between different outlets of the same level and sector—a finding that underscores the high complexity facing a potential contraceptive consumer when selecting a FP outlet in these urban and semiurban Nigerian health markets.^14^, ^15^, ^16^ The observed price differences between outlets are consistent with 2 hypotheses that likely operate jointly: first, outlets often stock a variety of brands within a single method type; variability could also exist because of heterogeneous pricing of the same products. Thus, it remains unclear to what extent most consumers “feel” differences in outlet-level minimum prices if their preferred brand is stocked and available at a consistent price. Furthermore, cost is only one of the many complex individual-, social-, and health systems-related factors influencing contraceptive behaviors. Nevertheless, consumers with the least ability to pay may face challenges for method initiation or resupply in the absence of widespread availability of a low-priced product option.

Using CM4FP's novel longitudinal outlet data, we were also able to explore changes in minimum prices by contraceptive method type within specific outlets over time. Although we observed relatively little heterogeneity in median prices over time in these local markets overall, cross-sectional “snapshots” of contraceptive prices mask the high prevalence of price changes at the outlet level. In fact, increases in outlet-level minimum prices were observed in most private nonfacility outlets when comparing the final with the second round of audit data. These findings may have important implications for young and unmarried populations, who often prefer private outlets but also may be affected by even small fluctuations in price. For the most price-sensitive consumers, increases in outlet-level minimum prices may have implications for likelihood of method initiation, use of a preferred method, and method resupply and continuation. Consumer experience and perceived quality of care may also be affected if consumers are required to “shop around” for lower prices at nonpreferred outlets.

This study has several strengths, including the longitudinal prospective design that allows for assessment of pricing changes over time; capture of the informal and nonfacility sector, including mobile vendors, which are a dominant source of FP commodities in Nigeria; and detailed product data on all available product brand–formulation combinations identified on the market. This study also has limitations. Because of the lack of concurrent longitudinal population-based data, we were unable to empirically examine how variability in contraceptive pricing, particularly that observed in the private sector, may affect contraceptive uptake and dynamics, including method selection, switching, continuation, and satisfaction. Additional limitations include the reliance on outlet self-reporting for pricing data where posted prices were not available. In addition, we lacked data on which products had posted (vs unposted) prices and utilization (and pricing ranges) of sliding-scale pricing systems, precluding a more formal assessment of informal pricing in these markets. Although we captured repeated observations at the outlet level, the relatively short duration of the study (3 quarterly audits) did not allow us to assess longer-term pricing trends or other market influences, such as possible seasonality. Finally, the CM4FP was a methodological pilot study in purposively sampled study sites; results are therefore not generalizable to broader subnational or national settings. The exclusion of rural study sites also precluded insights into rural contraceptive markets.

Longitudinal data collected from FP service delivery points offers unique insights into variability of pricing both between outlets of different types and within the same outlets over time in selected urban settings in Nigeria. Further research on consumers’ exposure to and experience of contraceptive price volatility; corresponding impact on access, method choice, initiation and continuation, and outlet selection; and the potential of market-shaping interventions to stabilize private sector prices in LMIC settings is warranted.
